# Research Progress on Astaxanthin Plus Exercise for the Prevention and Treatment of Knee Osteoarthritis: A Traditional Narrative Review

**DOI:** 10.3390/ijms27156773

**Published:** 2026-07-29

**Authors:** Rui Cheng, Wei Wu, Xiaomei Zu

**Affiliations:** 1 School of Exercise and Health, Shanghai University of Sports, Shanghai 200438, China; 2 School of Athletic Performance, Shanghai University of Sports, Shanghai 200438, China

**Keywords:** astaxanthin, osteoarthritis, antioxidant, anti-inflammatory, exercise

## Abstract

Knee osteoarthritis (KOA) is a degenerative joint disease characterized by inflammatory reactions and bone lesions within the joint, which is mainly caused by the degeneration of articular cartilage. This disease primarily affects middle-aged and elderly populations, especially those over 50 years old; however, a distinct trend of younger onset has been observed in recent years, which is closely associated with factors such as sports-related injuries and obesity. Astaxanthin (AST), a ketone carotenoid belonging to the xanthophyll family, exhibits extremely strong antioxidant and broad-spectrum anti-inflammatory activities with no obvious toxic and side effects due to its unique molecular structure, thus showing great potential in the prevention and treatment of KOA. This article reviews the research evidence and action mechanisms of AST in the prevention and treatment of KOA from multiple aspects, including alleviating joint pain, reducing cartilage structural damage, resisting oxidative stress, inhibiting inflammatory responses, and regulating cartilage metabolism and functional disorders, while exploring the key factors and signaling pathways involved. On this basis, a strategy combining AST with exercise intervention for the prevention and treatment of KOA is proposed, which provides a certain reference for the clinical treatment and scientific research of KOA.

## 1. Introduction

Knee osteoarthritis (KOA) is a degenerative joint disease characterized by the degeneration of articular cartilage, synovial inflammation, and subchondral bone remodeling. It is defined by the degeneration of articular cartilage, and its pathophysiology involves a vicious cycle of oxidative stress and chronic low-grade inflammation [1]. The exact causes of KOA remain unclear; the condition is thought to result from a combination of factors, including excessive physical strain, aging, genetic predisposition, sports injuries, and endocrine factors [1,2]. Pathological changes in KOA include progressive destruction of articular cartilage, subchondral bone sclerosis, and chronic synovial inflammation, with synovial inflammation considered the primary cause of joint damage and dysfunction [1]. At the same time, there are significant individual differences among patients with KOA, and there is a lack of precision in treatment tailored to different patients. Current strategies for treating KOA include weight management, exercise therapy, and physical therapy [1]. While offering the advantages of safety and the absence of side effects, these approaches rely on patient compliance; however, many patients find it difficult to maintain a long-term exercise regimen. Medication, on the other hand, is primarily used to alleviate pain and inflammation and plays a supportive role in the prevention and treatment of KOA. There is a lack of targeted treatments for KOA with low side effects. Against this backdrop, research has begun to focus on potential protective effects for the prevention and treatment of KOA. Studies have shown that astaxanthin (AST) can repair chondrocyte damage through various mechanisms, which may indirectly contribute to the prevention and treatment of KOA. Therefore, this article provides a review of the role of AST in the prevention and treatment of KOA to offer a research basis for clinical practice and scientific research.

## 2. Materials and Methods

This narrative review employed a comprehensive keyword search strategy to retrieve studies from the PubMed, Web of Science, Embase and China National Knowledge Infrastructure databases, covering the period from January 2013 to May 2026. The search terms employed in this study included “astaxanthin”, “osteoarthritis”, “knee osteoarthritis”, “exercise”, “physical activity”, “antioxidant”, “oxidative stress”, “anti-inflammatory”, “inflammation”.

A comprehensive selection of relevant studies was made, including original research articles and narrative as well as systematic reviews, to provide an overview of the most pertinent information on astaxanthin combined with exercise intervening in osteoarthritis via antioxidant and anti-inflammatory pathways. The research agenda was structured around two key pillars: original research and published reviews. Original research was prioritized for its direct relevance to the conceptual framework of this study, while systematic and narrative reviews were synthesized to provide comprehensive evidence syntheses. In instances where two studies reported analogous information, the more recent and exhaustive study was incorporated into the manuscript. In the course of this review, conference abstracts, case reports, unpublished grey literature, repeated documents and irrelevant papers focusing on unrelated disorders were excluded from consideration. While not exhaustive, this review synthesizes key findings from the collected literature to provide the most comprehensive overview possible of the antioxidant and anti-inflammatory mechanisms of astaxanthin combined with exercise against osteoarthritis.

Schematic figures were created using BioRender (https://app.biorender.com/, accessed on 7 May 2026).

## 3. Overview of AST

AST is primarily synthesized by microalgae, phytoplankton, yeast, and bacteria [3]. *Haematococcus pluvialis* is one of the best natural sources of AST and is currently known to be the organism with the highest AST content in nature [4]. AST has the molecular formula C_40_H_52_O_4_ and contains 13 conjugated double bonds, which give it unique chemical properties, molecular structure, and light-absorption characteristics, significantly enhancing its antioxidant capacity. However, synthetically produced AST differs slightly in molecular structure from natural AST, and its antioxidant activity is also significantly reduced [3]. In contrast to vitamin C and vitamin E, which lack conjugated double bonds, as well as conventional carotenoids such as β-carotene with only nine conjugated double bonds, AST exhibits significantly stronger free radical-scavenging capacity and superior inhibitory effects against oxidative stress. The multiple conjugated double bonds within the chemical structure of AST facilitate efficient free radical capture. Through electron donation, these conjugated double bonds react with free radicals to terminate the chain reactions initiated by free radicals in cells and tissues [4]. This structure enables it to more efficiently scavenge diverse reactive oxygen species (ROS), including singlet oxygen, superoxide anions, and hydroxyl radicals. Its singlet oxygen scavenging capacity is 200-fold higher than that of vitamin E and 4.3-fold higher than that of β-carotene, while its overall antioxidant activity is 6000-fold greater than that of vitamin C [5,6]. In contrast to water-soluble vitamin C and anthocyanins, AST is lipophilic [7]. This characteristic enables it to more readily penetrate cell membranes (lipid bilayers) of chondrocytes, synoviocytes, and other cells, and reach critical intracellular oxidative stress sites including mitochondria and the nucleus to scavenge intracellular ROS [8,9]. Conversely, water-soluble antioxidants face difficulty crossing cell membranes to exert their effects and thus only exert limited activity in the extracellular space or body fluids [10]. Furthermore, AST exerts pronounced anti-inflammatory and anti-apoptotic effects by downregulating the expression of multiple inflammatory mediators. Currently, clinical management of mild KOA lacks safe and long-term therapies that effectively alleviate symptoms, suppress inflammation and pain, and preserve joint function. AST shows no obvious toxic or adverse effects and is suitable for long-term application. Compared with other natural bioactive substances (e.g., curcumin and resveratrol), AST possesses a broader anti-inflammatory spectrum and offers more direct cytoprotection for chondrocytes. Current evidence suggests that the mechanisms underlying AST-mediated intervention in KOA include antioxidant and anti-inflammatory actions, inhibition of cartilage-degrading enzymes, and modulation of cellular metabolic signaling pathways.

### 3.1. Clinical Effects of AST

Masuma Tabassum et al. [11] conducted a randomized, double-blind, placebo-controlled trial to evaluate the effects of AST on pain, stiffness, physical function, and inflammatory markers in patients with moderate-to-severe KOA. Participants took 12 mg of AST capsules or a placebo orally every day for 8 weeks. The results revealed that the AST group exhibited significantly better improvements in pain, physical function, and Western Ontario and McMaster Universities Osteoarthritis Index (WOMAC) scores compared with the placebo group. Meanwhile, serum levels of high-sensitivity C-reactive protein (hsCRP) and interleukin-6 (IL-6) were markedly decreased. Furthermore, the incidence of adverse events was low, with no required treatment discontinuation. This study confirms that AST is safe and effective as an adjuvant therapy for patients with moderate-to-severe KOA. It can significantly ameliorate clinical symptoms and decrease inflammatory markers, demonstrating promising potential as a long-term therapeutic option. In addition, existing studies have indicated that the beneficial effects of AST generally emerge within 4 to 8 weeks. Accordingly, AST is more appropriate for long-term adjuvant therapy or health supplementation, rather than serving as an acute analgesic agent.

To explore the effects of AST-enriched krill oil in patients with mild to severe KOA, Welma Stonehouse et al. [12] designed a six-month multicenter, randomized, double-blind, placebo-controlled trial. Subjects in the intervention group received four FlexPrOMD^®^ krill oil capsules per day, corresponding to a total daily AST intake of 0.45 mg (450 μg). All capsules were administered in a single dose concomitantly with or right following meals. The study found that daily supplementation with krill oil significantly improved knee pain, stiffness and physical function at six months compared with placebo. As the key bioactive component of krill oil, AST exerts prominent pain-relieving effects under severe inflammatory conditions, while its efficacy remains limited in mild to moderate inflammation.

For patients with mild KOA, Singeun Kim et al. [13] evaluated the efficacy of FlexPrOMD^®^—a compound formulation containing krill oil, AST and hyaluronate—in individuals with Kellgren–Lawrence (KL) grade 1 or milder KOA. The AST group received one capsule orally once daily for 12 weeks. Each capsule of FlexPrOMD^®^ is formulated with 321 mg krill oil, 25–35 mg *Haematococcus pluvialis* extract (standardized to supply 2 mg AST), 33 mg hyaluronic acid, and 73–83 mg auxiliary excipients. The results indicated that FlexPrOMD^®^ yielded significant pain relief and showed a favorable trend in enhancing joint function. Accordingly, it can serve as a functional health food to assist the management of early-stage KOA in subclinical and borderline patients.

Randomized controlled trials have verified the favorable effects of AST on KOA, including pain alleviation, functional recovery and inflammatory mitigation. Nevertheless, its precise underlying mechanisms remain to be further elucidated.

### 3.2. AST Improves KOA via Multi-Target Mechanisms

#### 3.2.1. Inhibition of Inflammatory Responses and Cartilage Degradation

Min Hee Park utilized seven-week-old adult male rats and induced KOA via unilateral intra-articular injection of monoiodoacetic acid (MIA) [14]. MIA directly disrupts chondrocyte metabolism, triggers chondrocyte apoptosis, and impairs the dorsal root ganglia, replicating both the inflammatory and neuropathic pain phenotypes observed in advanced KOA. Animals in the AST treatment groups were administered FlexPrOMD^®^ orally. The low-dose group was treated with AST at ~0.108 mg/kg, while the medium- and high-dose groups received ~0.216 mg/kg and ~0.433 mg/kg, respectively. The sham-operated group was injected with an equivalent volume of normal saline, and all interventions lasted for 28 days following the establishment of the model. The results indicated that low- and medium-dose AST markedly restored hindlimb weight-bearing balance, alleviated cartilage structural destruction, surface irregularity and proteoglycan loss. Meanwhile, AST decreased serum levels of TNF-α, IL-1β and IL-6, and inhibited the mRNA expression of inducible Nitric Oxide Synthase (iNOS) and Cyclooxygenase-2 (COX-2) in joint tissues. Additionally, serum COMP and CTX-II levels were reduced, accompanied by downregulated mRNA expression of Matrix Metalloproteinase-2 (MMP-2) and Matrix Metalloproteinase-9 (MMP-9), thereby attenuating cartilage degradation. It has been demonstrated that oral administration of AST alleviates MIA-induced pain behaviors in KOA models, mitigates articular cartilage structural damage, suppresses systemic and local inflammatory responses, decreases the levels of cartilage degradation biomarkers, and inhibits the expression of matrix-degrading enzymes.

Li-Juan Huang et al. [15] constructed an in vivo osteoarthritis model using New Zealand rabbits through anterior cruciate ligament transection (ACLT), aiming to elucidate the modulatory effect of AST on the expression of chondral matrix-degrading enzymes. Each intra-articular injection consisted of 0.3 mL AST solution at a concentration of 50 μM. IL-1β, a central pro-inflammatory cytokine abundant in the synovial cavity of KOA, drives robust breakdown of the cartilage matrix and recapitulates the pathological inflammatory microenvironment characteristic of osteoarthritic lesions. Their experimental data revealed that AST markedly suppressed the mRNA levels of matrix metalloproteinase-1 (MMP-1), matrix metalloproteinase-3 (MMP-3), and matrix metalloproteinase-13 (MMP-13) in lesioned cartilage tissue. Integrating gross morphological evaluation and hematoxylin-eosin (H&E) histological staining analyses, obvious attenuation of cartilage destruction was detected in AST-treated groups. Collectively, these findings indicate that AST mitigates inflammatory injury to the cartilage matrix by transcriptionally repressing the synthesis of matrix-degrading enzymes.

Regarding the anti-inflammatory effects of AST, A.N.M. Mamun-Or-Rashid et al. [16] performed two parallel experiments with the murine monocyte/macrophage RAW264.7 cell line. Commercially available AST extracted from *Haematococcus pluvialis* was dissolved in dimethyl sulfoxide (DMSO), with the final solvent concentration adjusted to 0.2%. Two intervention concentrations (5 μg/mL and 50 μg/mL) were adopted throughout the assays. In the first experiment, cellular inflammation was triggered by Nε-carboxymethyl lysine-modified human serum albumin (CML-HSA). Briefly, cells were pretreated with AST for 24 h post-adherence, prior to 3 h continuous CML-HSA stimulation. In the second experiment, receptor activator of nuclear factor κB ligand (RANKL) was applied to induce osteoclast differentiation. Following 24 h of AST pretreatment after cell attachment, cells were subjected to sustained RANKL stimulation for 3 h. The results of the inflammation induction experiment demonstrated that the AST-treated group exhibited a reduction of at least twofold in the mRNA expression levels of TNF-α, IL-1β, IL-6, and iNOS; additionally, it significantly suppressed the expression of the Nuclear Factor of Activated T-cells, Cytoplasmic 1 (NFATc1), Cellular Fos (c-Fos), and Receptor for Advanced Glycation End-products (RAGE) genes. In the RANKL-induced osteoclast differentiation model, AST markedly inhibited Tartrate-Resistant Acid Phosphatase (TRAP) activity, multinucleated osteoclast formation, and F-actin ring formation, as well as downregulated the expression of osteoclast-related genes including TRAP, Cathepsin K (CTSK), MMP9, ATPase H+-transporting vacuolar-type (Atp6v) and NFATc1. Moreover, AST suppressed the activation of the NF-κB signaling pathway.

Experimental evidence confirms that AST, as a potential therapeutic agent for KOA, exerts comprehensive beneficial effects when formulated as a multi-component dietary supplement. As a multi-target natural compound, AST possesses promising prospects for further commercial development. Animal studies have demonstrated that oral AST administration markedly relieves pain-related behaviors in KOA model rats, attenuates cartilage damage and glycosaminoglycan loss, decreases the levels of multiple inflammatory factors, inhibits the expression of related genes, delays cartilage degradation, and suppresses the production of matrix-degrading enzymes [14]. Furthermore, AST can downregulate the gene expression of matrix-degrading enzymes in articular cartilage at the transcriptional level and alleviate cartilage degradation in experimental osteoarthritis [15]. In addition, AST markedly inhibits inflammatory responses in macrophages and aberrant osteoclast differentiation, downregulates the expression of inflammatory genes, and blocks the activation of key inflammatory signaling pathways [16].

#### 3.2.2. Protection of Chondrocytes Against Oxidative Stress

Chunhui Zhu et al. [17] constructed a rat knee osteoarthritis model via destabilization of the medial meniscus (DMM). In their study, combined intra-articular administration of recombinant R-spondin 2 (Rspo2, a key inflammatory mediator) and AST was performed, with dosages set at 20 μg/kg for Rspo2 and 20 mg/kg for AST, respectively. They evaluated joint injury, inflammatory status and cartilage quality, with a particular focus on macrophages, chondrocytes and their cell–cell interactions. One week after surgery, intra-articular injections of AST or Rspo2 were administered once weekly until the end of the study, and knee joint tissues were collected for subsequent analysis. The results demonstrated that AST significantly alleviated cartilage damage. Furthermore, the expression levels of inflammatory factors were remarkably reduced. In terms of histopathological staining of cartilage tissue, the AST group showed darker staining, indicating a significant protective effect on the cartilage matrix. It has been demonstrated that AST inhibits Rspo2-mediated inflammation in animal models, reduces M1 macrophage infiltration and activation, decreases the levels of pro-inflammatory factors in synovial fluid, preserves the integrity of the cartilage matrix, and delays cartilage degeneration. It effectively reverses the significant exacerbation of cartilage degeneration and inflammatory responses induced by Rspo2 injection, confirming its remarkable chondroprotective and anti-inflammatory effects in vivo, whose mechanisms are closely associated with the inhibition of the Rspo2/Wnt/β-catenin pathway.

Kai Sun et al. [18] disrupted cartilage homeostasis using IL-1β, TNF-α, and tert-butyl hydroperoxide (TBHP) to explore the regulatory effect of AST on the Nrf2 signaling pathway. Focusing on chondrocyte homeostasis and oxidative stress, this study emphasizes intracellular protective mechanisms and conducts research through multiple pathways. Primary mouse chondrocytes were isolated and cultured for in vitro assays, while DMM surgery was performed to establish an in vivo OA model. AST dissolved in a 20% Tween 20 solution was intra-articularly administered at a dosage of 20 mg/kg immediately after surgery, with injections delivered twice weekly over an 8-week consecutive treatment period. Experimental results demonstrated that AST facilitates the translocation of Nrf2 from the cytoplasm to the nucleus, activates its transcriptional activity, and strengthens antioxidant defense. Intra-articular administration of AST markedly enhances Nrf2 nuclear translocation in chondrocytes. Once activated, Nrf2 upregulates downstream antioxidant genes and mitigates oxidative stress, thereby inhibiting cartilage matrix degradation, alleviating chondrocyte apoptosis, and delaying KOA progression.

Wenwei Liang adopted human “C-28/I2 human chondrocyte cell line” (C28/I2) chondrocytes and established an oxidative stress injury model induced by tBHP [19]. tBHP, a stable organic peroxide, is widely used to mimic hydrogen peroxide-induced oxidative cellular damage. For the AST pretreatment group, cells were incubated with AST at a final concentration of 20 μM for 2 h prior to the induction of oxidative damage. Experimental results revealed that AST significantly reversed the decrease in C28/I2 cell viability induced by tBHP and reduced the chondrocyte apoptosis rate. Enzyme-Linked Immunosorbent Assay (ELISA) results demonstrated that AST suppressed the secretion of inflammatory cytokines (IL-6, IL-1β, TNF-α) in tBHP-stimulated C28/I2 cell culture supernatants. In terms of oxidative stress, AST markedly decreased the levels of ROS, Malondialdehyde (MDA) and Oxidized Glutathione (GSSG), restored the activities of Superoxide Dismutase (SOD) and Glutathione Peroxidase (GPX), and rebalanced cellular oxidative homeostasis. Moreover, AST significantly lowered tBHP-induced superoxide anion production and alleviated catalase depletion.

Collectively, these studies demonstrate that AST exerts a multi-target protective effect against KOA. It directly protects chondrocytes by activating the Nrf2 antioxidant pathway and modulates immune cell function via inhibition of the inflammatory mediator Rspo2, suggesting promising prospects for clinical translation and application [17,18].

Animal studies have demonstrated that oral AST administration alleviates KOA pain. As a dietary supplement, AST holds great potential as a therapeutic agent for the prevention and treatment of KOA. Furthermore, intra-articular injection of AST modulates key signaling pathways to confer antioxidant protection on chondrocytes and exert potent anti-inflammatory effects.

### 3.3. AST Administration Routes

AST presents limited oral bioavailability, ranging from 10% to 50%. This constraint mainly arises from its inherently low aqueous solubility and hampered transmembrane absorption by small intestinal epithelial cells. Naturally occurring AST, as well as that incorporated into most commercial supplements, predominantly exists as mono- and diester conjugates. Prior to intestinal absorption, these esterified derivatives require hydrolysis catalyzed by intestinal hydrolases to yield free AST. Its intestinal uptake is thought to rely predominantly on passive diffusion, independent of specific epithelial transporter proteins. Following oral ingestion, the elimination half-life of AST is approximately 15.9 ± 5.3 h [20]. Approaches for enhancing the oral bioavailability of AST are summarized as follows: (1) Lipid-based delivery systems (e.g., combinations of long-chain triglycerides and surfactants) can elevate bioavailability by 1.7–3.7-fold [20]. (2) Emulsifying agents represented by phospholipids contained in krill oil are capable of facilitating the intestinal absorption of AST [21]. Orally administered AST undergoes absorption across the gastrointestinal tract followed by systemic distribution. Upon reaching the articular cavity, its concentration is substantially diluted, resulting in markedly lower intra-articular drug levels relative to the initial administered dosage. In comparison, intra-articular AST injection circumvents gastrointestinal absorption barriers, directly delivering the active compound to the joint space and yielding superior local bioavailability. Comprehensive dosage data illustrating such disparities in absorption and therapeutic efficacy are summarized in the corresponding table. See Table 1.

Currently, the poor oral bioavailability of AST remains a major bottleneck limiting its clinical translation. Although intra-articular AST administration exhibits superior efficacy compared with conventional intra-articular agents in animal models, relevant human clinical trials are still lacking. Preclinical AST dosage conversion is commonly performed based on the body surface area method. According to standard preclinical pharmacological conversion coefficients, the equivalent human dose is approximately 12.3-fold that of a 20 g mouse, 6.2–6.3-fold that of a 200 g rat, and 3.1-fold that of a 1.5–2.0 kg rabbit [22,23]. A representative conversion formula for translating human clinical doses to rat doses is shown below [22]:

Rat dose = X mg/kg × 70 kg × 0.0018/0.2 kg = 6.3X mg/kg.

Furthermore, diverse delivery carriers substantially influence astaxanthin bioavailability.

## 4. The Protective Mechanism of AST Against KOA

The core pathological mechanism of KOA lies in the excessive catabolism of the cartilage matrix relative to anabolism, which ultimately results in cartilage degeneration. The initiation and progression of KOA are governed by the dysregulation of an intricate signaling pathway network. Owing to its multi-target characteristics, AST can intervene in multiple key signaling nodes simultaneously, thereby producing a synergistic chondroprotective effect. The therapeutic core of AST against KOA lies in restoring chondrocyte metabolic balance by regulating gene expression, signaling pathways, and non-coding RNAs to achieve multidimensional protective effects. Moreover, AST facilitates the synthesis of major extracellular matrix (ECM) components in chondrocytes, including type II collagen (COL2) and aggrecan, and upregulates the expression of anabolic markers. In addition, AST suppresses the expression of matrix metalloproteinases (MMPs), especially MMP-13, thereby attenuating cartilage matrix degradation and restoring joint homeostasis.

### 4.1. Inhibition of NF-κB and MAPK Pathways Exerts Synergistic Anti-Inflammatory Actions

The inflammatory response is an essential physiological defense mechanism whereby the body reacts to harmful stimuli, including injury and infection. However, excessive or persistent inflammatory responses act as a core pathological driver of multiple chronic diseases. At the molecular level, the initiation and progression of inflammatory responses are precisely regulated by an intricate network consisting of signaling pathways, transcription factors, and cellular interactions. In this network, the Nuclear Factor-kappa B (NF-κB) and Mitogen-Activated Protein Kinase (MAPK) signaling pathways function as central processors: they integrate various upstream signals and translate them into the expression of downstream pro-inflammatory genes, thereby initiating and amplifying the inflammatory cascade [24,25].

In mammalian cells, the NF-κB pathway usually exists in the form of heterodimers or homodimers, with the most prevalent being a dimer consisting of the p65 (RelA) and p50 subunits. This dimer is persistently and abnormally activated during the pathological progression of KOA, thereby contributing to the chronic inflammatory response and cartilage degeneration associated with the disease [26]. At rest, NF-κB (predominantly the p65/p50 heterodimer) binds to the inhibitory protein IκBα, sequesters in the cytoplasm, and remains transcriptionally inactive [27]. Upon stimulation by inflammatory mediators (e.g., IL-1α, TNF-α) or damage-associated molecular patterns (DAMPs), these stimuli are recognized by transmembrane receptors including Toll-like receptors (TLRs) and tumor necrosis factor receptors (TNFRs). Upon receptor activation, multiple adaptor proteins are recruited, ultimately triggering the activation of the key kinase complex TGF-β-activated kinase 1 (TAK1). Activated TAK1 further phosphorylates and activates the downstream IκB Kinase (IKK) complex, which in turn induces phosphorylation of IκBα and its subsequent degradation via the ubiquitination pathway [28,29,30]. The liberated NF-κB subsequently translocates into the nucleus and initiates the transcription of a panel of pro-inflammatory genes, including multiple cytokines (IL-6, TNF-α), chemokines, and MMPs [25].

In KOA chondrocytes, the NF-κB pathway regulates an extensive spectrum of downstream target genes. These include major catabolic factors such as matrix metalloproteinases (MMP-1, MMP-3, MMP-13), which directly degrade type II collagen and proteoglycans, along with pro-inflammatory mediators and apoptosis-associated proteins [26,31]. Therefore, aberrant activation of the NF-κB pathway is widely regarded as one of the master switches driving the pathological progression of KOA.

The MAPK family consists of three major parallel signaling cascades: the extracellular signal-regulated kinase (ERK), p38 MAPK, and c-JUN N-terminal kinase (JNK) pathways. As another pivotal signaling network activated in KOA, the MAPK pathway acts in close synergy with the NF-κB pathway [32]. The MAPK pathway can also be activated by stimuli including IL-1β and participates in regulating the expression of MMPs and inflammatory mediators.

Many inflammatory stimuli can concurrently activate the NF-κB and MAPK signaling pathways. The key upstream kinase TAK1 acts as a critical convergence node, capable of activating both the IKK complex and Mitogen-Activated Protein Kinase Kinases (MKKs), thereby synergistically triggering the downstream cascade reactions of the NF-κB and MAPK pathways [33]. Furthermore, the MAPK pathway can directly modulate NF-κB activity. Accumulated evidence has shown that activated p38 MAPK phosphorylates the p65 subunit of NF-κB, thereby further amplifying NF-κB-driven inflammatory responses [25,34]. In addition, the major downstream transcription factors of the NF-κB and MAPK pathways frequently function cooperatively. They co-bind to the promoters of numerous pro-inflammatory genes, thereby synergistically driving their robust expression [35,36]. This intricate crosstalk mechanism suggests that anti-inflammatory interventions simultaneously targeting key nodes of both pathways can yield more potent and efficient synergistic inhibition. AST is precisely such a molecule capable of acting on both pathways concurrently.

Studies have demonstrated that AST acts as a potent inhibitor of the NF-κB signaling pathway, which constitutes the core of its anti-inflammatory activity. Its underlying mechanism mainly suppresses upstream pathway activation, prevents inhibitor of kappa B alpha (IκBα) degradation, and inhibits the nuclear translocation of the p65 subunit. Research has shown that AST can suppress lipopolysaccharide (LPS)-induced Toll-like receptor 4 (TLR4)-mediated signaling pathways [37,38]. TLR4 serves as the primary receptor for LPS, a cell wall component of Gram-negative bacteria. Its activation represents the initial step in triggering robust inflammatory responses such as sepsis. Long-term or prophylactic administration of AST can downregulate TLR4 expression on the surface of immune cells including macrophages, thereby diminishing cellular sensitivity to LPS at the source [39,40]. Downstream of TLR4, Myeloid Differentiation Primary Response Gene 88 (MyD88) acts as a crucial adaptor protein that bridges the receptor to intracellular kinases. AST markedly downregulates MyD88 protein expression upon LPS stimulation, thereby further blocking downstream signal transduction [37,38]. Furthermore, AST pretreatment reverses the elevated phosphorylation level of Inhibitor of kappa B kinase β (IKKβ) induced by LPS and other pro-inflammatory stimulants [24,40]. Inhibition of IKKβ activity accordingly reduces the phosphorylation level of its direct substrate IκBα [24,41]. Unphosphorylated IκBα cannot be recognized and degraded by the ubiquitin-proteasome system, thus maintaining its stability within the cytoplasm [42]. By stabilizing IκBα, AST effectively sequesters NF-κB in the cytoplasm. The NF-κB p65/p50 dimer remains bound to IκBα, with its nuclear localization signal persistently masked. This prevents the p65 subunit from translocating into the nucleus [42,43,44].

**Figure 1:**
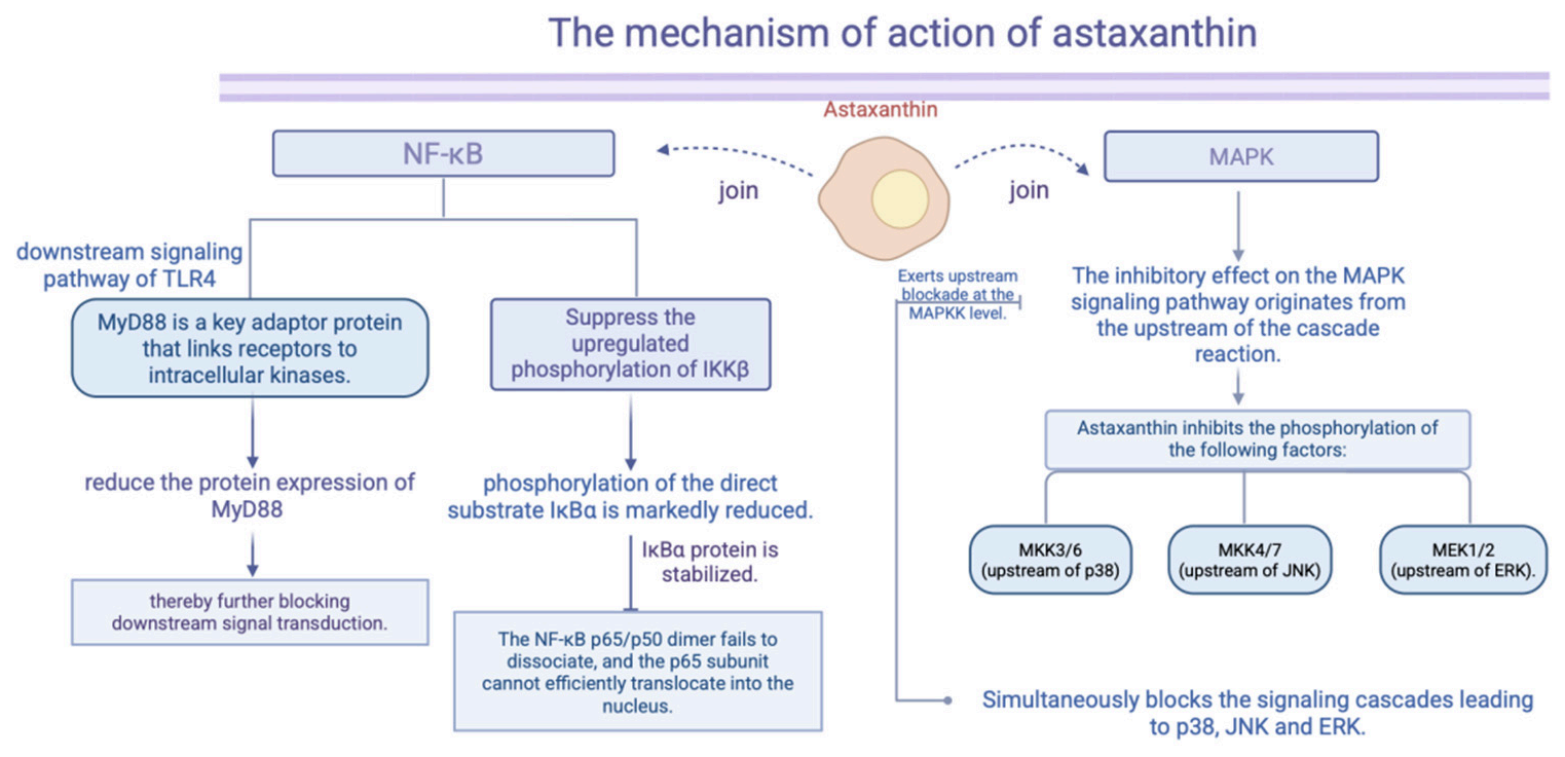
AST exerts anti-inflammatory effects by simultaneously targeting the NF-κB and MAPK signaling pathways. In the NF-κB pathway, AST blocks downstream signal transduction by reducing the expression of MyD88, an adaptor protein downstream of TLR4, and inhibits the phosphorylation of IKKβ to stabilize IκBα, thereby preventing the dissociation of the NF-κB p65/p50 dimer and the nuclear translocation of the p65 subunit to suppress the transcription of pro-inflammatory genes. In the MAPK pathway, AST acts at the MAPKK level to block upstream signaling cascades; it inhibits the phosphorylation of MKK3/6, MKK4/7 and MEK1/2, simultaneously attenuating the downstream activation of p38, JNK and ERK cascades, and ultimately achieves synergistic anti-inflammatory regulation via multiple pathways.

Similar to its inhibitory effect on the NF-κB pathway, AST suppresses the MAPK pathway at the early stage of the signaling cascade and markedly inhibits MAPK phosphorylation. As a member of the Mitogen-activated protein kinase kinase kinase (MAPKKK) family, TAK1 represents a potential key target of AST. AST suppresses the phosphorylation of MAP Kinase Kinase (MKK)3/6 (upstream of p38), MKK4/7 (upstream of JNK), and MAPK/ERK Kinase (MEK)1/2 (upstream of ERK), thereby blocking the MAPK signaling cascade at the MAPKK level [45]. It concurrently blocks the signaling cascades upstream of p38, JNK, and ERK, while directly suppressing the phosphorylation of these three MAPK members. This further attenuates subsequent NF-κB activation [46,47]. See Figure 1.

The unique advantage of AST lies in its capacity to synergistically regulate the NF-κB and MAPK pathways by precisely targeting the key nodes of their crosstalk. In addition to the shared upstream kinase TAK1, excessive reactive ROS can oxidize and activate multiple kinases such as IKK and MAPKKKs. This persistent activation sustains NF-κB and MAPK signaling, ultimately forming a vicious cycle between inflammation and oxidative stress [25,48]. Owing to its unique molecular structure, AST efficiently embeds within the cell membrane, scavenging intracellular and extracellular ROS to shield cells from oxidative damage and fundamentally neutralizing a crucial amplifier of inflammatory signaling. By reducing intracellular redox potential, AST establishes a low-reactivity cellular microenvironment that further facilitates the inhibition of downstream inflammatory signaling pathways [49,50].

In summary, AST fundamentally downregulates the expression of a range of downstream proteolytic enzymes and inflammatory mediators by directly targeting key steps of the NF-κB signaling pathway, particularly through inhibiting IκBα degradation and p65 nuclear translocation, as well as by synergistically suppressing the MAPK pathway. This constitutes one of the most critical and direct molecular mechanisms by which AST exerts preventive and therapeutic effects on KOA.

### 4.2. Activation of Nrf2/HO-1 Signaling Pathway to Enhance Endogenous Antioxidant Defense

Nuclear factor erythroid 2-related factor 2 (Nrf2) serves as a pivotal transcription factor governing cellular antioxidant responses. Under normal physiological conditions, Nrf2 is stably bound to its repressor, Kelch-like ECH-associated protein 1 (Keap1), in the cytoplasm. As a key component of the E3 ubiquitin ligase complex, Keap1 continuously ubiquitinates Nrf2, leading to its degradation by the proteasome [51]. When cells are subjected to oxidative stress or AST stimulation, the highly reactive cysteine residues of Keap1 undergo conformational alterations. AST directly interacts with these cysteine residues on Keap1, thereby suppressing Keap1-mediated Nrf2 ubiquitination [52,53]. The liberated Nrf2 rapidly translocates into the nucleus, binds to antioxidant response elements (AREs), and initiates the transcription of downstream phase II detoxification enzymes and antioxidant proteins [54,55]. Owing to the inhibition of Keap1-mediated Nrf2 degradation, Nrf2 protein gradually accumulates in the cytoplasm, leading to a marked increase in its concentration. Experiments have demonstrated that AST treatment significantly upregulates Nrf2 protein expression in chondrocytes, with this upregulation exhibiting a dose-dependent pattern [9]. Hemoglobin oxygenase-1 (HO-1) is an endoplasmic reticulum-localized antioxidant enzyme that breaks down hemoglobin to produce bilirubin, carbon monoxide, and iron ions, which exert antioxidant, anti-inflammatory, and anti-apoptotic effects. Therefore, the Nrf2/HO-1 pathway constitutes a key defense mechanism for cells against oxidative damage [56,57].

Studies have shown that AST molecules bind to specific pockets within the Keap1 protein, forming stable interactions with key amino acid residues through noncovalent forces including hydrogen bonds and van der Waals interactions [8,58]. For example, computational modeling studies have shown that AST can interact with residues such as Arginine (Arg)319, Arg415, and Glutamine (Gln)563 in Keap [58]. This interaction induces an overall conformational change in the Keap1–Nrf2 complex, disrupting the spatial configuration required for Keap1 to recognize and bind Nrf2, and thereby weakening the binding capacity of Keap1 toward Nrf2. This mechanism resembles steric interference, whereby AST functions as a molecular wedge inserted between Keap1 and Nrf2, ultimately facilitating Nrf2 dissociation [41,58]. This non-covalent and reversible binding mechanism may underpin the mild and sustained regulatory effect of AST under physiological concentrations.

**Figure 2:**
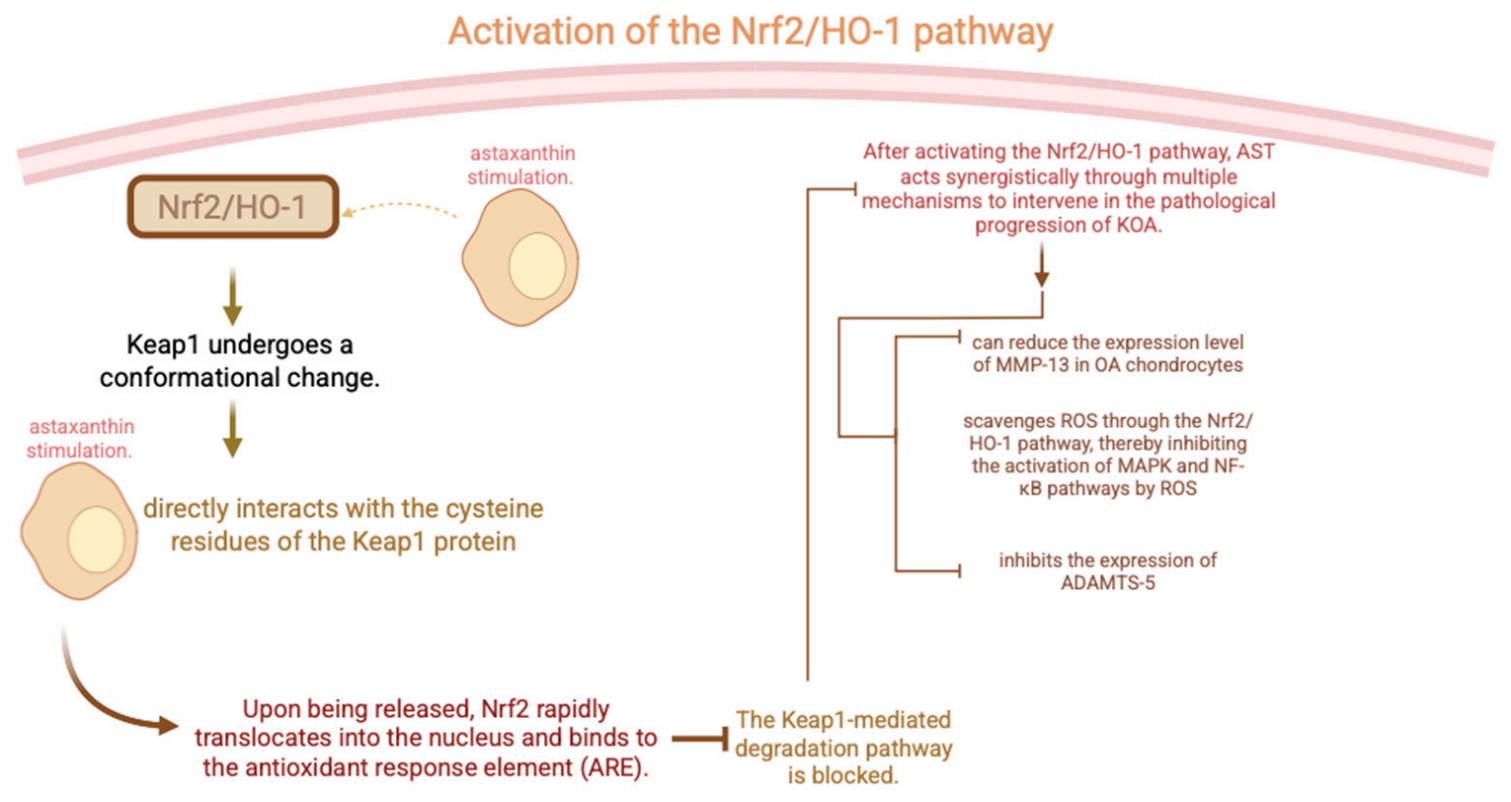
AST activates the Nrf2/HO-1 antioxidant pathway by directly interacting with cysteine residues of the Keap1 protein to induce its conformational change, which blocks the Keap1-mediated degradation of Nrf2. The dissociated Nrf2 rapidly translocates into the nucleus and binds to the antioxidant response element (ARE) to initiate downstream antioxidant responses. Following Nrf2/HO-1 pathway activation, AST intervenes in the pathological progression of KOA through multiple synergistic mechanisms: it down-regulates the expression of MMP-13 and ADAMTS-5 in osteoarthritic chondrocytes, scavenges ROS, and thereby inhibits ROS-induced over-activation of the MAPK and NF-κB pathways, ultimately exerting chondroprotective effects.

Upon Nrf2/HO-1 pathway activation, AST exerts synergistic regulatory effects on the pathological progression of KOA through multiple mechanisms. MMP-13 acts as a crucial cartilage-degrading enzyme and is primarily responsible for the breakdown of type II collagen. Studies have demonstrated that AST treatment can significantly downregulate the expression levels of MMP-13 in KOA chondrocytes [17,59]. ROS is a key mediator in the activation of the MAPK and NF-κB pathways. AST scavenges ROS through the Nrf2/HO-1 pathway, thereby inhibiting the activation of the MAPK and NF-κB pathways induced by ROS. Given that NF-κB serves as a key transcription factor regulating MMP-13 expression, the reduced activity of NF-κB ultimately leads to downregulated MMP-13 transcription. A Disintegrin-like And Metalloproteinase with Thrombospondin Motifs (ADAMTS)-5 is the primary proteoglycan-degrading enzyme. Similar to MMP-13, AST inhibits ADAMTS-5 expression by activating the Nrf2 signaling pathway, thereby reducing proteoglycan degradation and protecting the integrity of articular cartilage [18]. Experiments demonstrated that ADAMTS-5 expression was upregulated in response to inflammatory stimulation, while treatment with AST reversed this upregulation. When Nrf2 was knocked down, both basal and inflammation-induced ADAMTS-5 expression further increased, and the protective effect of AST was impaired—indicating that AST’s inhibition of ADAMTS-5 is highly dependent on the presence of Nrf2 [60,61,62]. See Figure 2.

In addition to its direct action on the Keap1-Nrf2 complex, AST may also indirectly promote Nrf2 pathway activation by regulating multiple upstream signaling pathways. The Nrf2 protein contains multiple serine (Ser) and threonine (Thr) residues that can be phosphorylated by various kinases; these phosphorylation modifications can modulate its affinity for Keap1, as well as its nuclear translocation efficiency and activity [63].

### 4.3. Regulation of Wnt/β-Catenin Pathway to Maintain Cartilage Matrix Integrity

One core mechanism by which AST prevents and treats KOA is the inhibition of the Rspo2-mediated Wnt/β-catenin signaling pathway, which facilitates the transition of chondrocytes from an inflammatory microenvironment toward restored metabolic homeostasis. In the pathological setting of KOA, this pathway exerts a critical catabolic effect. Rspo2 acts as a key activator of Wnt signaling; by amplifying pathway activity, it drives excessive chondrocyte differentiation and extracellular matrix degradation, thereby exacerbating articular damage [17]. Rspo2 binds to Leucine-rich repeat-containing G-protein-coupled receptor (LGR)4/5 and other receptors to suppress the degradation of Wnt receptors, consequently facilitating β-catenin stabilization and nuclear translocation. This process triggers excessive chondrocyte differentiation and extracellular matrix degradation, ultimately accelerating articular damage. In contrast, AST markedly downregulates Rspo2 expression, thereby restraining the aberrant activation of the Wnt/β-catenin signaling pathway.

**Figure 3:**
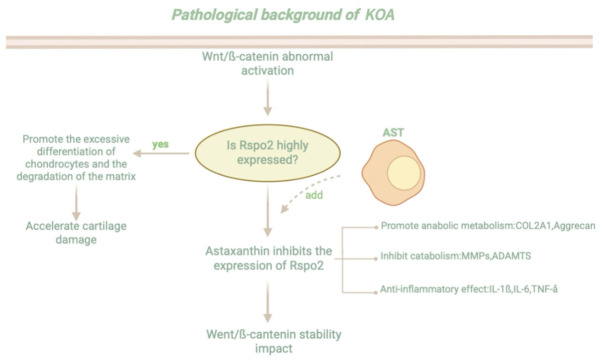
In the pathological progression of KOA, abnormal activation of the Wnt/β-catenin pathway induces high expression of Rspo2, which promotes excessive chondrocyte differentiation and extracellular matrix degradation, thereby accelerating cartilage damage. AST can suppress Rspo2 expression to regulate the stability of the Wnt/β-catenin pathway. It exerts chondroprotective effects by three synergistic approaches: promoting anabolic metabolism via upregulating COL2A1 and Aggrecan, inhibiting catabolic metabolism by downregulating MMPs and ADAMTS, and alleviating inflammatory responses through reducing the levels of pro-inflammatory cytokines including IL-1β, IL-6 and TNF-α, ultimately delaying OA-related cartilage degeneration.

Studies have demonstrated that the Wnt/β-catenin signaling pathway is aberrantly activated in the cartilage and synovium of KOA patients [64]. This excessive activation directly induces a catabolic phenotype in cartilage, upregulating the expression of catabolic genes including MMP-3, MMP-13, and ADAMTS-4/5, while concurrently downregulating the expression of anabolic genes such as Collagen type II alpha-1 chain (COL2A1), Aggrecan, and SRY-box transcription factor 9 (SOX9) [65,66,67,68]. In addition, it promotes chondrocyte hypertrophy and apoptosis via upregulation of Runt-related transcription factor 2 (Runx2) and Collagen type X alpha-1 chain (CoL10A1), while exacerbating local inflammatory responses [69,70]. Consequently, targeting the overactivated Wnt/β-catenin pathway has emerged as a key strategy in the development of drugs for KOA [21,70]. See Figure 3.

Anabolic processes are essential for the maintenance and repair of the cartilage matrix, and AST has been shown to upregulate the expression of anabolic markers in chondrocytes. Studies have demonstrated that AST treatment elevates the expression of COL2A1, the most abundant collagen component within the cartilage matrix, in chondrocytes [17]. This finding is consistent with the inhibitory effect on Wnt/β-catenin signaling, since excessive activation of this pathway suppresses COL2A1 expression [71]. As the predominant proteoglycan in cartilage, aggrecan binds abundant water molecules and endows cartilage with compressive resistance. AST has also been reported to upregulate aggrecan expression [17]. Studies on other Wnt inhibitors (e.g., sclerostin and strontium compounds) further support the notion that Wnt pathway inhibition enhances the expression of anabolic genes including aggrecan [71,72].

Another key mechanism underlying the chondroprotective effects of AST is the inhibition of the Wnt/β-catenin pathway. This enables AST to effectively suppress the expression of catabolic and inflammatory-related genes, including matrix metalloproteinases, ADAMTS family members, and pro-inflammatory cytokines [17,18,73]. In KOA pathogenesis, AST downregulates β-catenin levels via suppressing Rspo2, thereby directly blocking the aberrant activation of the Wnt/β-catenin pathway. This inhibits excessive β-catenin accumulation and nuclear translocation, which subsequently represses the expression of matrix-degrading enzymes including MMPs and ADAMTS, ultimately protecting the integrity of the cartilage matrix [17]. In particular, MMP-13 (collagenase-3) is the most pivotal enzyme responsible for type II collagen degradation. In KOA cartilage, aberrant upregulation of MMP-13 serves as a key driver of cartilage matrix degradation. Accumulating evidence has confirmed that MMP-13 acts as a critical downstream target gene of the Wnt/β-catenin signaling pathway [74,75]. Experimental data indicate that in an in vitro model, AST markedly downregulates MMP-13 expression in chondrocytes stimulated by IL-1β and other pro-inflammatory factors [17,18]. Within the ADAMTS family, ADAMTS-4 and ADAMTS-5 are the predominant enzymes that cleave the core protein of aggrecan. By suppressing the expression of these enzymes, AST effectively attenuates cartilage matrix degradation [18]. Aberrant Wnt signaling crosstalks with other inflammatory pathways. By inhibiting the Wnt/β-catenin pathway, AST reduces the secretion of key pro-inflammatory factors such as IL-1β, IL-6, and TNF-α in chondrocytes and macrophages; this anti-inflammatory effect exerts a synergistic action with its direct suppression of the NF-κB pathway [24,49]. Specifically, AST downregulates Rspo2 expression in chondrocytes and macrophages, thereby attenuating its positive amplification of Wnt signaling and ultimately reducing downstream β-catenin activity. This represents one of the most clearly defined molecular mechanisms by which AST modulates the Wnt pathway.

In addition to inhibiting Rspo2 at the upstream level, AST also modulates intracellular β-catenin stability, primarily targeting the key kinase Glycogen Synthase Kinase-3β (GSK3β). GSK3β activity is regulated by multiple signaling pathways, and a major inhibitory mechanism relies on phosphorylation at its Ser9 residue. When GSK3β is phosphorylated at the Ser9 residue by upstream kinases such as Akt, its kinase activity is suppressed. This impairs its ability to phosphorylate β-catenin, thereby promoting β-catenin stabilization [49,76,77]. Therefore, elevated levels of p-GSK3β (Ser9) generally indicate suppressed GSK3β activity and subsequent activation of the Wnt/β-catenin pathway. Current research suggests that AST may modulate β-catenin activity by regulating GSK-3β phosphorylation. Although direct evidence in chondrocytes remains limited, accumulating studies indicate that AST can regulate GSK-3β in other cell types. For example, studies have demonstrated that lipopolysaccharide (LPS)-induced inflammatory stimulation markedly increases intracellular p-GSK3β (Ser9) levels, indicating the inhibition of GSK3β activity. However, when cells were treated with AST at the same time, LPS-induced GSK3β phosphorylation was significantly suppressed [76]. This finding is crucial, as it demonstrates that AST can counteract the inhibitory effects of pathological stimuli such as inflammation on GSK3β activity. By preventing excessive phosphorylation at the GSK3β Ser9 site, AST effectively preserves GSK3β in an active state. Active GSK3β remains a functional component of the β-catenin degradation complex, efficiently phosphorylating β-catenin and targeting it for proteasomal degradation. This perfectly explains the significant reduction in total β-catenin protein levels in AST-treated cells. This mechanism constitutes the second core intracellular regulatory pathway through which AST modulates the Wnt signaling cascade. In KOA chondrocytes, AST-mediated regulation of GSK-3β activity and subsequent modulation of β-catenin stability are essential for elucidating its chondroprotective mechanism, yet definitive experimental evidence remains insufficient at present.

## 5. AST Combined with Exercise Intervention in KOA

The therapeutic mechanisms of AST in KOA include alleviating joint pain, attenuating cartilage structural damage, resisting oxidative stress, inhibiting inflammatory responses, and regulating abnormal cartilage metabolism. At the molecular level, AST as one of the most potent natural carotenoids protects chondrocytes against free radical damage by activating the Nrf2 pathway and enhancing cellular antioxidant defense systems, including the upregulation of SOD activity, thereby exerting antioxidant protective effects [18]. In terms of inflammatory inhibition, AST markedly suppresses the expression of pro-inflammatory factors including TNF-α, IL-1β, and IL-6, blocks the NF-κB and MAPK signaling pathways, and attenuates inflammatory levels in the articular cavity [18,73,78]. In terms of inhibiting cartilage degradation and regulating cellular metabolism and apoptosis, AST downregulates the expression of matrix metalloproteinases, especially MMP-1, MMP-3, and MMP-13, thereby alleviating cartilage matrix degradation [78,79]. In addition, by upregulating the Silent Information Regulator 1 (SIRT1) signaling pathway, AST also inhibits the Wnt/β-catenin pathway and ferroptosis, thereby preserving the mitochondrial structure of chondrocytes and suppressing chondrocyte apoptosis.

Relying solely on AST-related medications is unlikely to fully restore joint function. KOA is essentially characterized by the wear, degeneration, and aging of articular cartilage. It is most prevalent in middle-aged and elderly populations, as well as in obese individuals. With aging, articular cartilage loses its elasticity and exhibits reduced self-repair capacity. Furthermore, obesity increases the load on knee and hip joints, thereby accelerating cartilage wear and tear. Therefore, the “AST + exercise” combination is regarded as the gold standard for treating KOA. Specifically, AST serves as a powerful adjuvant to exercise therapy for KOA. By leveraging the unique properties of AST, it can effectively reduce oxidative damage induced by exercise, promote the recovery of damaged chondrocytes, and further enhance muscle adaptability to better support joint function [78,80].

Walking is the safest and most effective form of aerobic exercise. It is advisable to walk on flat, soft ground such as treadmills or lawns to alleviate joint strain. Furthermore, the buoyancy of water can markedly reduce joint loading; accordingly, aquatic exercises such as swimming are highly recommended for patients with severe pain or excessive body weight. Studies have shown that aquatic exercise exerts prominent effects on pain relief and functional improvement [80,81]. In terms of exercise intensity, patients supplemented with AST may tolerate slightly higher exercise intensity than conventional recommendations, while remaining within the moderate range to avoid excessive oxidative stress—defined as light sweating with the ability to maintain conversation. A minimum of 150 min of moderate exercise per week is recommended, optimally divided into daily sessions of 30–45 min [81,82].

Strength training should adhere to low-impact and high-frequency principles, aiming primarily to strengthen the quadriceps and gluteus medius while reducing mechanical stress on the knee and hip joints [81,82]. Patients can perform seated straight-leg raises while sitting upright on a chair. Fully straighten and lift the knee, hold the position for several seconds, and then slowly lower the leg. This exercise mainly targets the quadriceps at the anterior thigh and is suitable for patients with obvious knee pain. Additionally, patients can complete resistance band glute bridges in a supine position: bend the knees, lift the hips to the maximum height, contract the gluteal muscles tightly, so as to strengthen the gluteal and core muscles. For strength training in the prevention and treatment of KOA, moderate-to-high-intensity exercise has been proven to substantially alleviate pain and enhance muscle strength. For patients with stable symptoms and well-controlled joint pain, training intensity should be set at 60–85% of one-repetition maximum (1RM), with 2–3 sets per session. In contrast, for patients who suffer from marked pain and severe joint discomfort during high-intensity exercise, intensity should be restricted to a very low load of 20–30% of 1RM [83].

It is recommended to ingest AST post-exercise in combination with omega-3-rich krill oil or hyaluronic acid to enhance antioxidant capacity and joint lubrication, alleviate oxidative stress, and accelerate chondrocyte repair [21]. AST is fat-soluble; administration with meals can enhance its absorption efficiency. Nevertheless, AST exhibits poor oral bioavailability, wherein merely 10–50% of the administered dosage undergoes intestinal absorption. This phenomenon is primarily attributed to the compound’s inherently low aqueous solubility and inherent constraints on intestinal uptake. Specifically, its limited solubility within the gastrointestinal lumen, coupled with weak miscibility in digestive fluids and its propensity to form complexes with choline and dietary lipids, collectively hinders its transmembrane absorption across intestinal epithelia [84,85,86]. The oral bioavailability of astaxanthin differs substantially across diverse formulations. Internal comparative assays performed by raw material supplier AstaMAZ on commercially accessible astaxanthin preparations reveal that emulsions deliver the optimal bioavailability at 74.83%, a value markedly superior to that of alternative dosage forms. For reference, the bioavailability of microcapsules and tablets stands at 47.16% and 45.07%, respectively. Hence, the rational selection of astaxanthin formulations ought to be matched to distinct exercise modalities [87]. See Table 2 for detailed recommendations.

## 6. Conclusions

As a global degenerative joint disorder, KOA is pathologically characterized by chronic low-grade inflammation, progressive cartilage degradation, and disrupted extracellular matrix metabolic balance. At present, there are no available disease-modifying drugs that can effectively slow or reverse disease progression. Under such circumstances, exploring bioactive compounds with multi-target regulatory effects from natural products has become a promising research hotspot. AST, a potent natural carotenoid, has attracted extensive attention in chronic disease research owing to its prominent antioxidant and anti-inflammatory properties. This review summarizes and evaluates the current evidence on AST in the prevention and treatment of KOA, aiming to elucidate its core functional mechanisms and assess its clinical translational potential. Based on existing studies, this paper establishes a solid theoretical and experimental basis for the chondroprotective effects of AST, covering the maintenance of articular cartilage structural integrity and the regulation of extracellular matrix metabolic homeostasis. Second, the beneficial effects of AST on KOA are attributed to its capacity to concurrently modulate multiple interrelated pathogenic signaling pathways, highlighting its unique advantages in multi-target synergistic regulation. However, at the basic research level, most current evidence is derived from rodent models, whose pathological processes differ from those of chronic progressive KOA in humans. In addition, variations in AST dosage, administration routes and formulations across existing studies have hindered the establishment of a standardized optimal intervention regimen. At the clinical research level, existing RCTs are generally limited by small sample sizes and short follow-up durations, with a lack of large-scale, multicenter, long-term evidence regarding efficacy and safety [21]. Furthermore, the bioavailability of AST in humans, its actual concentration distribution within joint tissues, and whether its efficacy varies across joint sites or patient subgroups (e.g., individuals with different degrees of obesity and inflammatory status) remain largely unclear. This indicates that future research on AST for the prevention and treatment of KOA should prioritize further elucidating its underlying molecular mechanisms. Such investigations are of great significance for promoting the clinical application of AST in preserving knee structural integrity and achieving long-term symptomatic relief, as well as for standardizing its optimal dosage and formulation regimens. In summary, current evidence strongly supports the therapeutic potential of AST in the prevention and treatment of KOA. Its multi-mechanism synergistic effects are well suited to target the complex, multifactorial pathogenesis of KOA. Although translating this promising natural molecule into a widely adopted clinical intervention still relies on the accumulation of high-level evidence-based medical data and advances in formulation technology, existing research has undoubtedly established a solid scientific basis and delineated a clear roadmap for its translational progression from basic laboratory investigation to clinical application.

## Figures and Tables

**Table 1:** The Effects of Different Routes and Doses of AST on KOA.

Study Type	Study Population	Route of Administration	Dose	Primary Efficacy Endpoint	Ref.
Clinical Trials in Humans	Patients with moderate-to-severe KOA	Oral	12 mg/day	Relieves pain and stiffness, improves joint function, reduces inflammatory factors, is well-tolerated, and is suitable for long-term use as an adjunct therapy	[11]
	Patients with mild to severe KOA		0.45 mg/day (450 μg, 4 FlexPrOMD^®^ tablets)	Long-term supplementation helps improve joint symptoms; provides stronger pain relief for severe inflammation, but has limited effectiveness for mild to moderate inflammation	[12]
	Patients with mild KOA (KL1 or below)		2 mg/day (FlexPrOMD^®^)	Significant pain relief and mild improvement in joint function; suitable for health maintenance in individuals with early-stage subclinical conditions	[13]
Animal Studies	MIA-Induced Rat KOA Model		0.108 mg/kg and 0.216 mg/kg	Anti-inflammatory; inhibits matrix-degrading enzymes; reduces the loss of chondroitin sulfate; restores weight-bearing balance	[14]
	ACLT-Induced KOA Model in New Zealand White Rabbits	Intra-articular Injection	50 μM, 0.3 mL per dose	Transcription-repressed matrix metalloproteinases mitigate structural damage to cartilage	[15]
	DMM-Induced KOA Model in Rats		20 mg/kg (Once a week)	Inhibits Rspo2-mediated inflammation, reduces macrophage infiltration, and protects the cartilage matrix	[16]
	DMM Mouse KOA Model + Primary Chondrocytes		20 mg/kg (Twice a week for 8 weeks)	Inhibits Rspo2-mediated inflammation, reduces macrophage infiltration, and protects the cartilage matrix	[17]
In vitro cell experiments	RAW264.7 Mouse Monocytes and Macrophages	Cell culture medium	5 μg/mL, 50 μg/mL	Inhibits macrophage inflammation, blocks osteoclast differentiation, and downregulates NF-κB pathway activity	[18]
	C28/I2 Human Chondrocytes		20 μM	Antioxidant, anti-chondrocyte apoptosis, reduces oxidative byproducts, and restores antioxidant enzyme activity	[19]

**Table 2:** Different Exercises and the Appropriate Astaxanthin Formulation to Take.

Intervention Category	Specific Category	Detailed Parameters	Target Population	AST Supplement	Improvement Indicators	Ref.
Aerobic Exercise	Walking on Flat Ground	At least 150 min of moderate-intensity exercise per week; 30–45 min per day	Most patients with KOA	Standard Soft Gel Capsules	Decreases in Visual Analogue Scale (VAS) pain scores and WOMAC joint function scores, and increases in range of motion (ROM)	[80,81,82,87]
	Water Exercises (Swimming/Water Walking)	2–3 times a week, 30–45 min each time	Patients with severe pain, obese patients	Lotion	Decreased mechanical load on the joints and lower VAS pain scores	
Strength Training Strength Training	Seated Straight- Leg Raise	Stable symptoms: 60–85% of 1RM, 2–3 sets per session; Significant pain: 20–30% of 1RM	People with significant knee pain	Soft Gel Capsules + Krill Oil Combination Formulation	Increased quadriceps strength and knee joint stability	[81,82,83,87]
	Resistance Band Glute Bridge		All patients with KOA	Soft Gel Capsules + Krill Oil Combination Formulation	Reduced mechanical stress on the hip joint and increased core stability	

## Data Availability

No new data were created or analyzed in this study. Data sharing is not applicable to this article.

## Abbreviations

OA: Osteoarthritis, AST: Astaxanthin, ROS: reactive oxygen species, TNF-α: Tumor Necrosis Factor-alpha, IL-1β: Interleukin-1 β, Nrf2: Nuclear factor erythroid 2-related factor 2, hsCRP: high-sensitivity C-reactive protein, IL-6: interleukin-6, KL: Kellgren-Lawrence, WOMAC: Western Ontario and McMaster Universities Osteoarthritis Index, MIA: monoiodoacetic acid, MMP-1: Matrix Metalloproteinase-1, MMP-2: Matrix Metalloproteinase-2, MMP-3: Matrix Metalloproteinase-3, MMP-9: Matrix Metalloproteinase-9, MMP-13: Matrix Metalloproteinase-13, ACLT: anterior cruciate ligament transection, iNOS: inducible Nitric Oxide Synthase, COX-2: Cyclooxygenase-2, mRNA: messenger Ribonucleic Acid, ERK1/2: Extracellular-Regulated Kinase 1/2, DMSO: *Haematococcus pluvialis*, CML-HSA: Nε-(Carboxymethyl)lysine-human serum albumin, RANKL: Receptor Activator of Nuclear Factor-κB Ligand, NFATc1: Nuclear Factor of Activated T-cells, Cytoplasmic 1, c-Fos: Cellular Fos, RAGE: Receptor for Advanced Glycation End-products, TRAP: Tartrate-Resistant Acid Phosphatase, CTSK: Cathepsin K, Atp6v: ATPase H+-transporting vacuolar-type, DMM: destabilization of the medial meniscus, Rspo2: R-spondin-2, TBHP: tert-butyl hydroperoxide, H2O2: hydrogen peroxide, C28/I2: C-28/I2 human chondrocyte cell line, ELISA: Enzyme-Linked Immunosorbent Assay, MDA: Malondialdehyde, GSSG: Oxidized Glutathione, SOD: Superoxide Dismutase, GPX: Glutathione Peroxidase, ECM: extracellular matrix, COL2: including type II collagen, MMPs: matrix metalloproteinases, NF-κB: Nuclear Factor-kappa B, MAPK: Mitogen-Activated Protein Kinase, DAMPs: damage-associated molecular patterns, TLRs: Toll-like receptors, TNFRs: tumor necrosis factor receptors, TAK1: TGF-β-activated kinase 1, ERK: extracellular signal-regulated kinase, JNK: c-JUN N-terminal kinase, IKK: IκB Kinase, MKKs: Mitogen-Activated Protein Kinase Kinases, IκBα: inhibitor of kappa B alpha, LPS: lipopolysaccharide, TLR4: Toll-like receptor 4, MyD88: Myeloid Differentiation Primary Response Gene 88, IKKβ: Inhibitor of kappa B kinase β, MAPKKK: Mitogen-activated protein kinase kinase kinase, MKK: MAP Kinase Kinase, MEK: MAPK/ERK Kinase, Keap1: Kelch-like ECH-associated protein 1, AREs: ntioxidant response elements, HO-1: Hemoglobin oxygenase-1, Arg: Arginine, Gln: Glutamine, ADAMTS: A Disintegrin-like And Metalloproteinase with Thrombospondin Motifs, Ser: serine, Thr: threonine, LGR: Leucine-rich repeat-containing G-protein-coupled receptor, COL2A1: Collagen type II alpha-1 chain, SOX9: SRY-box transcription factor 9, Runx2: Runt-related transcription factor 2, CoL10A1: Collagen type X alpha-1 chain, GSK3β: Glycogen Synthase Kinase-3β, SIRT1: Silent Information Regulator 1, 1RM: one-repetition maximum.
